# A consensus-based elastic matching algorithm for mapping recall fixations onto encoding fixations in the looking-at-nothing paradigm

**DOI:** 10.3758/s13428-020-01513-1

**Published:** 2021-03-22

**Authors:** Xi Wang, Kenneth Holmqvist, Marc Alexa

**Affiliations:** 1 TU Berlin, Berlin, Germany; 2 Nicolaus Copernicus University, Torun, Poland; 3 University of the Free State, Bloemfontein, South Africa; 4 Regensburg University, Regensburg, Germany

**Keywords:** Episodic memory, Visual importance, Looking at nothing

## Abstract

We present an algorithmic method for aligning recall fixations with encoding fixations, to be used in looking-at-nothing paradigms that either record recall eye movements during silence or want to speed up data analysis with recordings of recall data during speech. The algorithm utilizes a novel consensus-based elastic matching algorithm to estimate which encoding fixations correspond to later recall fixations. This is not a scanpath comparison method, as fixation sequence order is ignored and only position configurations are used. The algorithm has three internal parameters and is reasonable stable over a wide range of parameter values. We then evaluate the performance of our algorithm by investigating whether the recalled objects identified by the algorithm correspond with independent assessments of what objects in the image are marked as subjectively important. Our results show that the mapped recall fixations align well with important regions of the images. This result is exemplified in four groups of use cases: to investigate the roles of low-level visual features, faces, signs and text, and people of different sizes, in recall of encoded scenes. The plots from these examples corroborate the finding that the algorithm aligns recall fixations with the most likely important regions in the images. Examples also illustrate how the algorithm can differentiate between image objects that have been fixated during silent recall vs those objects that have not been visually attended, even though they were fixated during encoding.

## Introduction

In the looking-at-nothing paradigm, participants are asked to inspect a scene, while their eye-movements are being recorded, and then to recall the contents of the same scene from memory while looking at an empty display. The researcher using this paradigm compares the fixations from the inspection trial with those of the subsequent memory retrieval trial, to draw conclusions of which scene elements are prioritized in memory recall or how the visual episodic memory is organized.

The looking-at-nothing effect was first demonstrated by Moore (1903), and later research established that in the absence of other visual features (i.e. while looking at nothing), the motion of the eyes is reminiscent of the gaze pattern while looking at the original stimulus (Johansson et al., 2006; Laeng et al., 2014). As part of this line of research, Noton and Stark (1971b) and Noton and Stark (1971a) have reported to have found that, to examine an image, humans tend to repeat a stereotyped, personal scan-path. However, there has been no later support for the idea that the sequence of fixations is reiterated and/or stored in memory (Williams & Castelhano, 2019; Kowler, 2011; Findlay & Gilchrist, 2003). All recent attempts at replication have found that fixations during recall of the stimulus reveal the *location* of objects (Ferreira et al., 2008; Martarelli et al., 2017), but not necessarily reinstate the sequences (Martarelli & Mast, 2013; Gurtner et al., 2019).

It has also been found that participants with a good spatial imagery ability make fewer recall eye movements, while participants with a poor ability make more and wider eye movements (Johansson et al., 2012). A number of studies (Johansson et al., 2012; Johansson & Johansson, 2014; Laeng et al., 2014; Scholz et al., 2015; Bochynska & Laeng, 2015; Pathman & Ghetti, 2015; Scholz et al., 2016) have shown that eye movements during recall play a *functional* role in memory retrieval. Laeng and Teodorescu (2002) showed that inhibiting eye motion, by asking observers to maintain fixation on a central point during encoding, led to reduced eye motion during recall, and inhibiting eye motion during recall led to degraded recall performance. de Vito et al., (2014) confirmed that inhibiting eye motion during recall decreases memory performance. Moreover, attending to regions that have been previously looked at before has been linked to imagery vividness (Laeng and Teodorescu, 2002), change detection performance (Olsen et al., 2014), memory accuracy (Laeng et al., 2014; Scholz et al., 2016) and recognition accuracy (Chan et al., 2011), further suggesting that eye movements during looking at nothing correlate to what has been encoded in memory.

There is, however, a fundamental limitation to the looking-at-nothing paradigm: The locations of fixations during recall exhibit a significant local displacement, i.e. the spatial reproduction of fixation positions contains error. This deformation of the imagery space has been consistently reported in imagery literature and involves shrinking, translation and not making any eye-movements at all (Johansson et al., 2006; Johansson et al., 2012; Laeng et al., 2014). To overcome the obstacle of shrinkage, instead of using natural images, most previous studies employed single face images (Chan et al., 2011; Henderson et al., 2005) or grid-based stimuli (Martarelli & Mast, 2013; Scholz et al., 2016; Laeng et al., 2014; Johansson & Johansson, 2014), for which area-of-interest (AOI) methods are sufficient to find the correspondences between encoding fixations and recall fixations. However, for complex stimuli such as photographic images, visual features are irregularly distributed and rigid area-of-interest methods (commonly used to analyse gaze data) very often fail to handle the displacements in recall locations, forcing researchers to perform time-consuming manual coding, often using spoken language as a mediator to link recall fixations with spoken scene content (Johansson et al., 2006, e.g).

In this paper, we propose a new method to computationally match fixations while viewing the original image to fixations from spontaneously recalling the same image from visual episodic memory. In order to match fixations during recall to fixations during exploration, we therefore need to compute a mapping. After applying the mapping, we retain fixations from the exploration sequence that are close enough to a fixation in the relocated recall. A threshold on the distance between fixations in the exploration sequence from fixations in the recall allows us to steer the distance criteria and to control the amount of image content being considered as recalled (more detailed in Elastic consensus method for matching recall fixations onto encoding fixations).

We then validate the matching algorithm by checking whether the matched fixation positions coincide with separate judgments by participants clicking in the images on what they consider the most important scene regions. If there is a strong correlation between clicking and the areas highlighted by the matching algorithm, we would have a measure of what participants prioritized in the recall from short-term visual episodic memory. Please note that we do not investigate visual episodic memory here, and do not make any theoretical claims as to what is encoded in memory, nor which models could describe retrieval prioritization best. Our interest in this paper is only to develop a method for empirically researching memory recall using the looking-at-nothing paradigm. We will use the term ‘recall’ for ‘retrieval of memory content’ during looking-at-nothing even though the recalled information is not spoken but only exhibited through gaze.

## Data acquisition of eye movements during encoding and recall

We first collected eye movement data during the encoding and immediate subsequent recall of randomly selected photographic image content. The complete data set for pairs of exploration and recall eye movements can be found on the http://cybertron.cg.tu-berlin.de/xiwang/mental_imagery/em.html.^1^

### Method

#### Participants

We recruited 28 participants for our experiment (mean age = 26, SD = 4, 9 female). All reported normal or corrected-to-normal vision. Importantly, all participants were kept naive with respect to the aim of the study, i.e. they had no knowledge about the *purpose* of recalling the presented images from memory against a neutral background. All collection of data has been approved by the local Ethics Committee at the faculty IV of Technische Universität Berlin in compliance with the Guidelines of the German Research Foundation on Ethical Conduct for Research involving humans. Participants were informed about the procedure before giving their written consent and could stop the experiment at any time. Their time was compensated and all data were used anonymously.

#### Apparatus

The data collection was conducted in a dark and quiet room. A 24-inch display (0.52*m* × 0.32*m*) with a resolution of 1920 × 1200 pixels was in front of the observer at a distance of 0.7*m*. We used an EyeLink1000 desktop mount system (SR Research, Canada) to record the eye movements at a sampling rate of 1000*Hz*. A chin and forehead rest was used for stabilization. All data were recorded during a binocular viewing condition, but only the movements of the dominant eye were recorded.

#### Stimuli

We used images from the MIT data set (Judd et al., 2012), which also include eye-movement data. In order to make sure that we will have sufficient spatial variation in their eye-movement data, we calculated the 2D entropy of fixation positions for each image in the complete data set, which ranged from 0.358 to 0.587, which was deemed sufficient, and led us to select 100 natural images randomly. This set includes both indoor and outdoor scenes of various complexity and exhibits a large variation in both theme and composition. Since our main focus is to develop and test the matching algorithm, rather than studying specific memory effects, we chose not to control the images in any other way. All images were presented at the centre of the display in their original size with the largest dimension being 1024 pixels.

#### Procedure

**Fig. 1:**
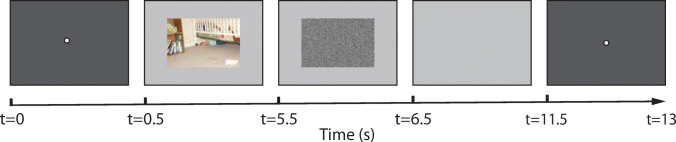
Recording paradigm used in data collection 1. Each stimulus was first viewed for 5s and observers were asked to immediately recall the image

We first explained the task in detail. The whole data recording consisted of 100 trials. The details of the presentation in one trial were: First, the screen was black with a white fixation dot in the centre (1^∘^ visual angle) for 0.5 seconds (500 ms). Then the image was presented for a duration of 5 seconds. Observers were instructed to freely explore each image in order to later be able to recognize it in a separate memory probe. After the image had been offset, white noise was shown for 1 second to suppress any after-image. Then the screen was set to neutral grey for 5 seconds, during which observers had been asked to *immediately* recall the image from memory. After that the screen turned black for 1.5 seconds before the procedure is repeated for the next image (see an illustration in Fig. 1).

Every participant first had a trial run of 10 other images at the beginning. The order of the 100 images were randomized and then divided into five blocks. Each block of 20 trials started with a standard 9-point calibration procedure. We repeated the calibration until the average accuracy reported in the following validation was below 0.5^∘^ and no validation point had an error larger than 1.0^∘^. After a successful calibration, 20 trials were performed. This procedure required roughly 5 minutes. Participants were allowed to take a break of arbitrary duration after each block.

The whole data acquisition lasted about one hour for each participant. At the end of the data collection, participants were shown 10 images, half of which were part of the 100 stimuli used in the previous trials. Images were presented one after the other in a randomized order, and participants had to decide if the images were among the 100 previously presented to them. These recognition data were not used, but only served to masquerade the data collection as a memory task, motivating them to actively explore the images after they initially hear about the memory probe.

### Data processing and analysis

We first analyzed the eye movement statistics from all 28 observers for the encoding and recall phases. We applied the EyeLink event detection algorithms with standard parameter settings (with saccade velocity threshold set to 35^∘^/*sec* and saccade acceleration threshold set to 9500^∘^/*sec*/*sec*) to detect fixations and saccades.

**Fig. 2:**
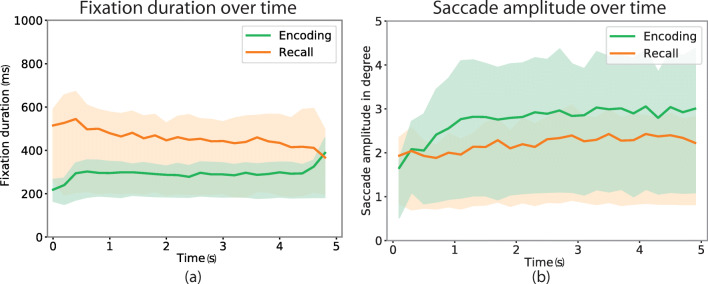
Fixation durations and saccade amplitudes are plotted as a function of the trial progression. The comparison of fixation durations over time in encoding and recall is shown in **a** and the comparison of saccade amplitudes over time in encoding and recall is shown in **b**. The curves indicate the mean durations and amplitudes and the range within one standard deviation are depicted in green for fixations and saccades from the encoding phase and orange for fixations and saccades from recall

During encoding the median and mean number of fixations was 16 (SD= 2.8) during encoding, while during recall the median and mean number was 11 (SD= 3.6). The fewer fixations in recall had a correspondingly longer duration (M = 452.2 ms, SD = 308.0 ms) than fixations in encoding (M = 278.0 ms, SD= 73.4 ms), as depicted in Fig. 2a. Fixation durations are plotted as a function of the trial progression.

#### Fixation durations over time

Durations of encoding fixations were lower during the initial 0.5s, and then constant throughout each trial, while the durations of fixations from recall got shorter over time. Buswell (1935) and (Unema et al.,, 2005, Figures 2 and 4) propose that short initial fixations reflect a period of ambient processing before focal viewing takes over with longer fixations. In contrast, the initial fixations of the recall data were the longest of their kind, which could suggest that when recalling from memory, there was no ambient phase, because the overview may already be accessible in memory.

#### Saccade amplitudes over time

Surprisingly, we did not find any ambient/focal effect in the encoding saccades, but rather its opposite, short saccades in the initial half second. We also note that recall saccades were about the same size throughout the trials as depicted in Fig. 2b.

#### Gaze data from encoding

The encoding fixations from all participants for a single image can be summarized in a spatial histogram (a so-called gaze density map, which is also called the *encoding map* in later sections), commonly plotted as a *heat map* for the image. We computed the spatial histograms for our data and for the corresponding publicly available MIT-data. Following Judd et al., (2012) we removed the first centre fixation from each sequence and applied a Gaussian filter with a kernel size equivalent to 1 degree of visual angle. The heat maps resulting from the publicly available data and the heat maps from the exploration phases in our experiment were very similar (mean Pearson’s correlation coefficient (CC) = 0.766, SD= 0.115).

#### Gaze data from recall

**Fig. 3:**
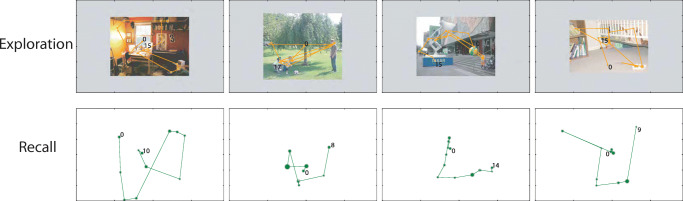
Pairs of exploration and recall fixation sequences from four observers. Fixation sequences during exploration are shown in the first row overlaid on top of the image stimuli. Corresponding fixations during recall are shown in the second row. The temporal order of each sequence is indicated by the numbers and consecutive fixations are connected by line segments. Fixation duration is reflected by circle size. Notice that fixations in recall are distorted relative to image features and there are often no clear correspondences between fixations during exploration and recall

Compared to encoding sequences, recall sequences had fewer but longer fixations (see Figure 2a). For some of the 100 photos, the correspondence between fixations during encoding and recall was very clear. However, for the majority, while fixations from recall roughly resembled the maps from the encoding phase, recall fixations were more constrained towards the centre and typically fail to *exactly* correspond with features in the original image that participants would have remembered during recall. We also found that the temporal order was in general not preserved (see Fig. 3 for some examples). This is consistent with previous results in imagery research (Johansson et al., 2012).

Some observers tended to stall during recall. They stopped moving their eyes, leading to fixations that are unlikely corresponding to image content. For 5 out of the 28 participants, the number of fixations in recall was less than half compared to encoding. One participant reported after the experiment that he changed his strategy through the experiment and only recalled the single most interesting element of the image.

#### Quality of individual recall data

**Fig. 4:**
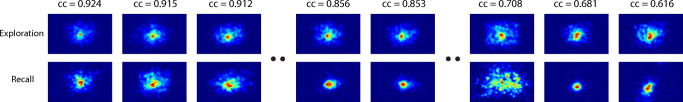
The similarity between the encoding and recall gaze density maps for each of the 28 participants aggregated over the 100 stimulus images. The 28 participants are ranked according to the correlation between the two cumulative gaze density maps (max 0.924; median 0.856; min 0.616). Two different types of inconsistencies appear in the least similar pairs: the distributions of recall fixations are either peaked at one point (the last two columns) or spread randomly (the third from the last column)

As expected from previous studies, some participants developed certain strategies during the data collection that distort the data in the sense that their eye movements during recall were minimal. Typical distortion included a low number of fixations clustered around the centre (central bias), or fixations that were randomly spread over the stimulus area that cannot be used to reliably identify locations of scene elements corresponding to one of the fixations during encoding. This naturally presents a challenge in the matching task. For this reason, we evaluated the quality of each data set in terms of the degree by which participants spontaneously made looking-at-nothing eye-movements. The median correlation between encoding and recall gaze density maps was *CC* = 0.856, when they were aggregated over all 100 stimulus images, for each participant. A few exemplary aggregated gaze density maps from encoding and recall, and the resulting correlation coefficients (CC) are illustrated in Fig. 4. Previous studies have only presented these distortions qualitatively (Johansson et al., 2006, e.g.), while others have used other measures of gaze dispersion (Johansson et al., 2012, e.g.), which makes quantitative comparison with earlier work difficult.

## Collection of clicking data

In our second data collection, participants were asked to to identify the (subjectively) most important scene element by clicking at its position after being briefly exposed to a stimulus. Clicking has previously been used to determine important areas (Nyström and Holmqvist, 2008), as an alternative to asking people to judge selected image patches for importance (as in Henderson and Hayes (2017)).

We will then compare the clicked areas with recall fixations, to validate that the matched recall objects from our algorithm correspond to positions judged as important by our participants. In this, we follow studies that show how the gist of a scene can be perceived in a single glance within as little as hundred milliseconds (Potter & Levy, 1969; Biederman et al., 1974; Oliva & Torralba, 2006; Castelhano & Henderson, 2008).

### Method

#### Participants

A group of 21 participants (6 female, mean age = 24) were recruited and their participation was compensated. All participants had normal or corrected-to-normal vision and none of them had taken part in previous data collection. Consent forms were signed before the experiment which allow us to use their data.

#### Apparatus

The apparatus setup was identical to that used in previous data collection. Observers’ eye movements were tracked with a standard EyeLink 1000 in remote mode.

#### Stimuli and design

**Fig. 5:**
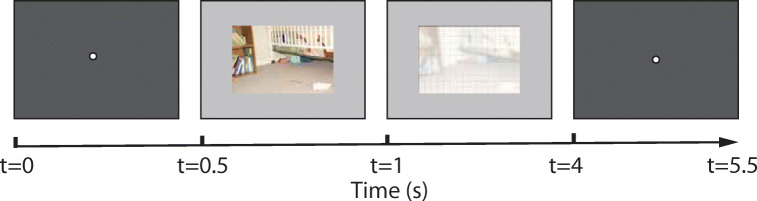
Recording paradigm used in data collection 2. After a brief exposure of image stimulus, observers were asked to click at the most important scene element

The same set of 100 images were used as visual stimuli. In order to help observers better locate scene elements, grids were overlaid over the images as inconspicuously as possible. Smoothed, semi-transparent images were displayed during clicking. High spatial frequencies were removed from the images and remaining low frequencies functioned as a reminder of the visual content. A Gaussian kernel with radius of 10 was used for smoothing and the alpha transparent blending value was set to 0.3 (an example is shown in Fig. 5).

#### Procedure

The procedure for each trial is illustrated in Fig. 5. Similarly to the previous data acquisition procedure, each trial started with a black screen with a white fixation circle at its centre, followed by a brief encoding phase where an image stimulus was displayed for 500 ms, which is more than enough time to perceive the important elements in each image. Observers were instructed to ‘select the most important part of the image’. Subsequently, observers were asked to ‘click at that selected position’. The amount of time given for clicking was 3s in each trial.

### Data processing

**Fig. 6:**
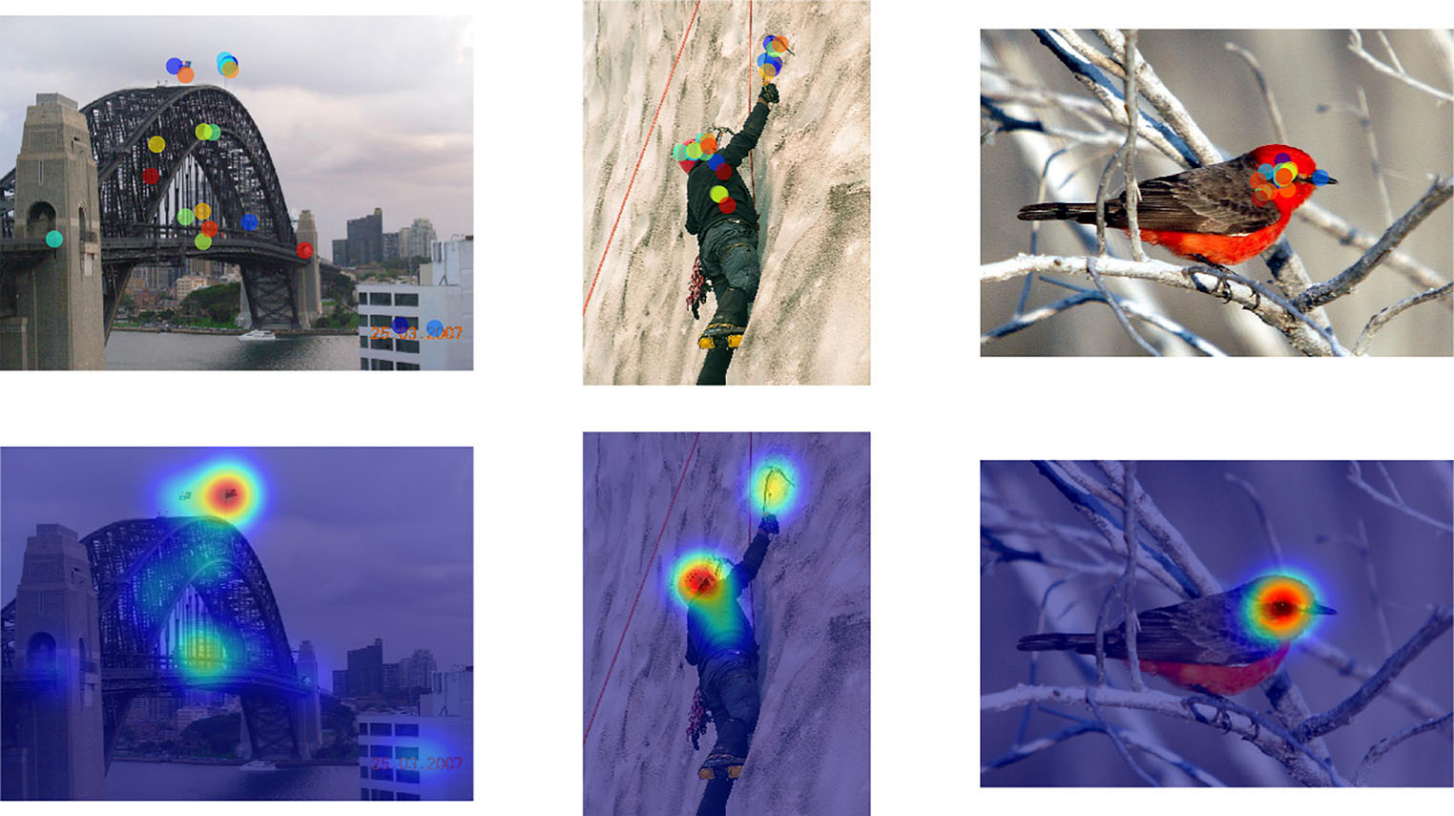
Examples of clicking results where agreement of clicking positions among all participants increases from left to right. First row shows clicked positions after the instruction to click what is important. Below it, heat maps of the same click data

We used 2093 out of 2100 trials, for which observers clicked within the given time. The average click latency was 1.63*s* measured from the moment when the image got blurred. This may also indicate that the short preview of 500 ms was enough to perceive the scene and the removal of high-frequency information may help to decrease the influence of low-level salient features on where people click. Figure 6 shows three examples of the data where heat maps for click data were smoothed with a Gaussian kernel of 2^∘^ of visual angle. These heat maps are called the *clicking maps* in later sections.

## Elastic consensus method for matching recall fixations onto encoding fixations

In the first data collection, participants were asked to immediately recall the previously seen image in front of an empty screen, while we measured their eye-movements in line with the looking-at-nothing paradigm. We will now present an algorithm that determines which scene positions in the original image correspond to the looking-at-nothing (recall) fixations.

### The principles of relocation mapping

The challenge in establishing this mapping is that spatial locations of the fixations in recall are distorted relative to the locations of features in the image. Fixations during looking-at-nothing contain a sizable deformation, and the inaccuracy in each fixation may accumulate over time. For this reason, we consider the operation to relocate the recall fixations to their proper position as a composition of a global rigid transformation (due to a lack of reference frame) and local deformation (due to local inaccuracy and propagated errors). We speculate that without this relocation, several recalled elements remain unmatched, causing significant but unwarranted changes to the mapping.

**Fig. 7:**
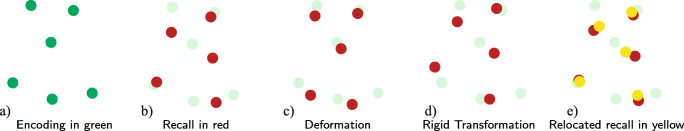
Example of the location mismatch between fixations in encoding and recall. Fixations from encoding are shown in green **a**; recall fixations are subset of the fixations during encoding and are shown in red **b**. Their displacement involves deformation **c** and global rigid transformation **d**, which are used to model different types of distortion observed in recall data. After the relocation mapping, recall fixations are transformed to the yellow locations **e**. Deformation and rigid transformation are computed in an iterative manner, avoiding convergence at local minima. Please refer to The principles of relocation mapping for more details

It is important to realise that the method does not aim to match nearest neighbours. Deformation provides greater freedom in mapping to neighbouring encoding fixations, while the rigid transformation aims to preserve the overall layout of recall fixations. Figure 7 provides an illustration of how these two forces interact in the mapping principle: The six green points in a) depict a configuration of fixations during encoding, of which only five are being recalled in looking-at-nothing fixations (b, red points). We may think of the locations as being displaced by the rigid transformation in d) and the (local) deformation in c). The deformation contains no global rigid transformation in the sense that moving or rotating the fixations would not make the sum of the squared distances to the matched fixations in encoding any smaller. Note that matching fixations in recall and encoding cannot be found by simply considering their distances (as in the nearest-neighbour algorithm; Anderson et al., (2015)), as such matching will not take into account the relative structure within recall fixations (see Fig. 3 for some examples).

When data are corrupted by global transformation and deformation, it is common to approximate the effects by minimizing the squared distances between matched data points. This presents a chicken and egg problem: The estimated mapping is intended to improve the matching, while the matching is needed to estimate the mapping. A common solution is to start with a first guess about the matching, then compute the transformation, and based on the transformation make a better guess about the matching, and so on until the method converges. We take our starting point in the Iterated Closest Point method (ICP) (Besl and McKay, 1992) to compute the global rigid transformation to match the two partially overlapping point sets (encoding vs recall fixations).

Our method extends ICP in two important ways: First, rather than using a closest point matching, it matches fixations in recall to *consensus* locations in the encoding data. Since there are fewer recall fixations than encoding fixations, we suggest computing a *consensus location* for each recall fixation. The consensus location is a weighted average of the fixation locations in encoding where the the weights decay exponentially with distance. This would give us three different cases to accommodate.

1. One fixation in recall maps to exactly one fixation from encoding. The consensus calculation will then have the effect when the single fixation in the encoding sequence is close while the others are far away, since the closest location will receive a large weight, while the others get relatively small weights, so that the consensus location will be the matching fixation.
2. One fixation in recall maps to several close fixations from encoding, i.e. the observer recalls just one scene element that generated several fixations during encoding. In case several fixations from encoding are close, all of them receive largely equal weights, and the consensus location is the weighted centre position of this set of fixations. This could correspond to situations where participants carefully inspect the eyes, nose and mouth of a face, which correspond to many fixations during encoding, but during looking at nothing only the face area is recalled / fixated.
3. A fixation in recall may have no corresponding fixation in encoding (i.e. because the fixation is unrelated to the process of recalling, the spatial error is too large to be rectified, or a non-fixated scene element is recalled). All fixations receive little weight, and the consensus location is roughly where the recall fixation already is.

Figure 7e) shows the consensus locations for the situation in b) with recall fixations are placed at the consensus locations after applying the relocation mapping. The mathematical modeling of the computation of consensus locations is explained in Computing the relocation of recall fixations. It can be controlled by a parameter *w*_*p*_, measured in visual angle, which could be interpreted as the distance of fixations that contribute significantly to the weighted averaging procedure. We set the parameter to *w*_*p*_ = 2^∘^ and discuss this choice in Effect on the relocation mapping of the parameters *w*_*p*_ and *w*_*d*_.

The second extension of ICP is that, rather than computing a global rigid transformation, our method is based on a *elastic mapping* that has a controllable overall deviation from a global rigid transformation. In such a way, a global transformation offers a reference frame for the overall fixation distribution during looking at nothing while a local transformation provides the flexibility to adjust the variant local distortions. Allowing elastic transformation overcomes the problem depicted in Fig. 7d).

However, some global rigidity needs to be preserved, i.e. the positions should not deform arbitrarily, as fixations while looking at nothing are not arbitrary but correlated to the spatial layout of a mental imagery during recall (Johansson et al., 2006, e.g.). This is important, as we would otherwise always match all recall fixations to some fixations in encoding. For example, if we allowed arbitrary scale, it would always be possible to scale the set of recall fixations to a single point and then match it to one of the fixations in encoding. To avoid such degenerated solutions where recall fixations are mapped to the centroid of all encoding fixations, it is important to restrict the mapping to preserve the global structure.

Table 1 lists the definitions of the relevant maps for clarity. Essentially the leftover map equals the encoding map minus the matched encoding map. The matched encoding map shares similarities with the relocated recall map, however, the matched encoding map does not equal the relocated recall map.

**Table 1:** Overview of relevant fixation and gaze density maps used in the algorithm

Encoding map	Gaze density map generated from *original encoding* fixations
Matched encoding map	Gaze density map generated from *matched encoding* fixations,
	which corresponds to the recalled content
	also called map of remembered things
Leftover map	Gaze density map generated from encoding fixations
	that are *not* matched to any relocated recall fixations,
	which correspond to the un-prioritized / forgotten scene elements
	also called map of non-prioritised objects or map of forgotten things
Recall map	Gaze density map generated from *original recall* fixations
Relocated recall map	Gaze density map generated from *relocated recall* fixations
Clicking map	Density map generated from click data

### Computing the relocation of recall fixations

In this section, we describe the computation of a relocation mapping $D: \mathbb {R}^{2} \mapsto \mathbb {R}^{2}$ from the current positions of the recall fixations to the desired ones. The global rigidity of the mapping is controlled by a parameter *w*_*d*_, measured in visual angle, which describes the distance of points that may be transformed by two rigid transformations that differ significantly. If *w*_*d*_ is large, fixations are transformed by very similar rigid transformations, restricting the deformation; if it is small, fixations are transformed by independent rigid transformations, allowing large deformation. We set the parameter to *w*_*d*_ = 10^∘^ and discuss this choice in Effect on the relocation mapping of the parameters *w*_*p*_ and *w*_*d*_.

The necessary steps to compute the relocation mapping are given in pseudocode below. Once the mapping is computed, we simply use distance as the sole criterion when a fixation in encoding should be considered as recalled, namely if there is a mapped fixation in recall that is closer than the matching radius *𝜖*.

Let $\mathbf {p}_{i} \in \mathbb {R}^{2}$ be the positions of the fixations in the encoding sequence and $\mathbf {r}_{j} \in \mathbb {R}^{2}$ the positions of fixations in the recall sequence (in a common coordinate system). We wish to compute a relocation $D: \mathbb {R}^{2} \mapsto \mathbb {R}^{2}$ that is applied to the recall locations **r**_*j*_ with the aim to align the data with the fixation positions during encoding.

We need two ingredients for computing the relocation: partial matching and elastic mapping. For the elastic mapping, we suggest Moving Least Squares (MLS) (Levin, 1998). Here, we use this framework applied to rigid transformations, i.e. local rigid transformations are fitted using weighted least squares. This approach has become popular in geometric modelling where it is usually derived as minimizing the deviation of the mapping from being locally isometric (Schaefer et al., 2006; Sorkine & Alexa, 2007; Chao et al., 2010).

We model the relocation *D* as a rigid transformation that varies smoothly over space:

1 $$ D(\mathbf{x}) = \mathbf{R}_{\mathbf{x}} \mathbf{x} + \mathbf{t}_{\mathbf{x}}.  $$

The subscript **x** indicates that rotation and translation vary (smoothly) with the location **x** in the plane. They are computed by solving a spatially weighted orthogonal Procrustes problem Gower and Dijksterhuis (2004), where the weights depend on the distances of the points to **x**. Assume the desired position for **r**_*j*_ is the position **q**_*j*_, then **R**_**x**_,**t**_**x**_ are computed by solving

2 $$ \arg{}\min_{\mathbf{R_{\mathbf{x}}}^{\mathsf{T}}\mathbf{R_{\mathbf{x}}} = \mathbf{I}, \mathbf{t}_{\mathbf{x}}} {\sum}_{j} \theta(\| \mathbf{x} - \mathbf{r}_{j}\|) \| \mathbf{R}_{\mathbf{x}}\mathbf{r}_{j} + \mathbf{t}_{\mathbf{x}} - \mathbf{q}_{j} \|_{2}^{2}.  $$

Here, the weight function *𝜃* should be smoothly decaying with increasing distance. We use the common choice

3 $$ \theta_{d}(x) = e^{-\frac{x^{2}}{{w_{d}^{2}}}},  $$

which gives us control over the amount of elasticity in the mapping with the parameter *w*_*d*_. The minimization can be solved directly using the singular value decomposition (SVD), see Sorkine-Hornung and Rabinovich (2016) for an accessible derivation.

Note that for computing the mapping we simply assume the desired positions **q**_*j*_ were given. We compute them as the distance weighted centroid of encoding fixations:

4 $$ \mathbf{q}_{j} = \frac{{\sum}_{i} \theta_{p}(\| D(\mathbf{r}_{j}) - \mathbf{p}_{i} \|) \mathbf{p}_{i}}{{\sum}_{i} \theta_{p}(\| D(\mathbf{r}_{j}) - \mathbf{p}_{i} \|)},  $$

where *𝜃*_*p*_(*d*) quickly decreases such that points further away are receiving relatively insignificant contribution.

**Fig. 8:**
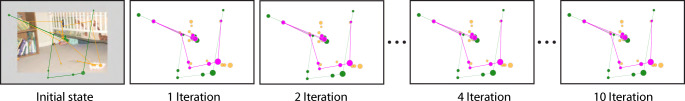
Iterations of the elastic relocation mapping algorithm. The initial state shows two fixation sets of encoding (orange) and recall (green). The size of each circle corresponds to the duration of each fixation. Pink circles are the deformed recall fixations after each iteration. Note that the matching converges quickly after just a few iterations

Note that we are considering the distances of the fixations **p**_*i*_ to the *relocated* locations of the fixations in the recall sequence. This means that setting the target locations depends on the relocation mapping and computing the relocation mapping depends on the target locations. Consequently, we alternate the two steps as shown in Algorithm 1.^2^ We start this process with *D* being the identity. Then we compute the desired positions **q**_*j*_ as explained above. The procedure converges after very few iterations (see Fig. 8). Note that the relocation *D* needs to be evaluated only in the location **r**_*j*_. This means for the next step we only need to compute **R**_**x**_ and **t**_**x**_ for **x** = **r**_*j*_.

**Figure:**
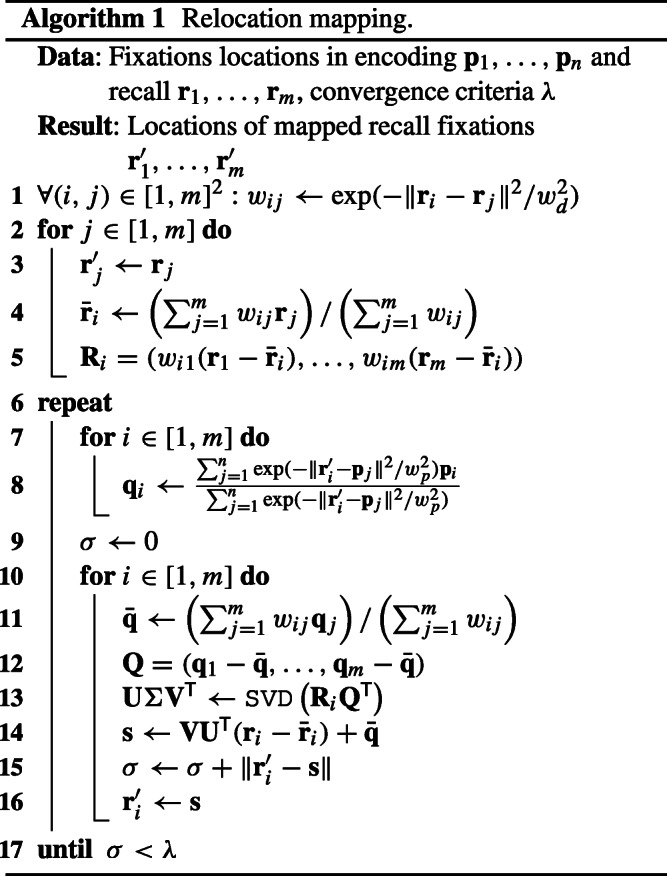

### Effect on the relocation mapping of the parameters *w*_*p*_ and *w*_*d*_

**Fig. 9:**
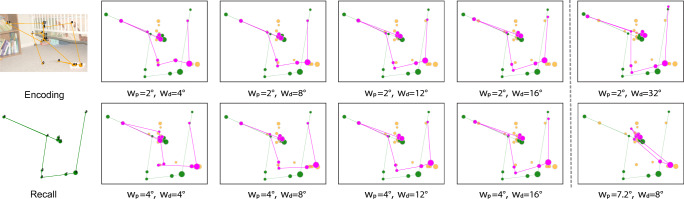
Matching results for a range of values for *w*_*p*_ and *w*_*d*_. Original recall fixations (in green) are matched to fixations during encoding (in yellow) and relocated recall fixations are shown in pink. Matching between recall and encoding is stable across all parameters, except in the rightmost column, which shows cases of undesired mapping for extreme parameter settings. More encoding fixations are considered for matching with a larger *w*_*p*_ while the relocation mapping is more rigid with a larger *w*_*d*_

The outcomes of the process for relocating recall fixations were stable across a wide range of parameters, as Fig. 9 illustrates. We found that the radius for matching recall fixations to encoding fixations *w*_*p*_ should be chosen in the range of 2^∘^− 4^∘^ of visual angle. This means we expected that matching fixations were usually not separated by more than twice this amount. To limit the amount of the relocation in the mapping we had tried values of *w*_*d*_ ∈ [4^∘^,16^∘^]. Based on trial and error, we had settled for *w*_*p*_ = 2^∘^ and *w*_*d*_ = 10^∘^. We applied the relocation process to each pair (encoding/recall) of fixation sequences for all images. Then we applied a matching using *𝜖* = 1^∘^, such that an encoding fixation was matched only if there was a corresponding relocated recall fixation within 1^∘^ visual angle. This was a rather strict setting, leaving many encoding fixations unmatched. We optioned for a small *𝜖* for stronger effects.

**Fig. 10:**
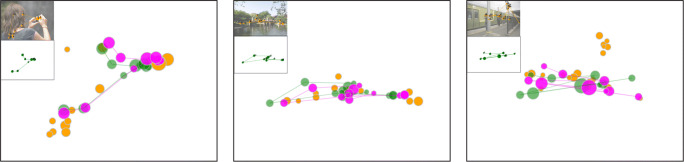
The relocation result on three different examples. Parameters *w*_*p*_ is set to 2^∘^ and *w*_*d*_ to 10^∘^. Fixations during encoding are depicted in yellow and recall fixations in green. Mapped recall fixations are shown in pink. Encoding fixation sequences overlaid with the stimulus and the corresponding original recall fixation sequences are illustrated at the top-left corners

Figure 10 shows three examples of relocated recall fixations based on the corresponding encoding fixations, using this parameter setting.

### Mapping relocated recall fixations onto encoding fixations using different matching radii *𝜖*

Our algorithm in the previous section moves original recall fixations in the stimulus space to generate a map of relocated recall fixations. Next, we will match - based on distance - each relocated recall fixation to one or more original encoding fixation(s). After we have established the mapping, we select those encoding fixations that were matched to relocated recall fixations. Because there are almost always fewer fixations in recall, there will be *leftover* encoding fixations that - under the assumptions of this algorithm - correspond to scene content that was never expressed in recall fixations (in laymen terms; forgotten, not prioritized, disregarded).

**Fig. 11:**
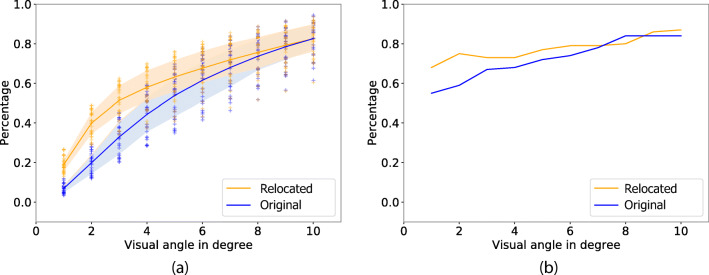
In **a**, the reduction rate as a function of matching radius *𝜖* ∈ [1^∘^,10^∘^]. The original recall fixations are shown in blue. Because they match with fewer of the encoding fixations, reduction rate is higher for the original recall fixations. Recall fixations that have been relocated are more likely to be mapped onto an encoding fixation (yellow), and therefore there will be fewer leftover encoding fixations and a lower reduction rate for relocated recall fixations, in particular when *𝜖* is small. **b** This figure shows for what proportion of the 100 images the peak of the gaze density map for encoding fixations is within 4^∘^ of the peak of the gaze density map for the matched encoding fixations based on the relocated recall fixations, as a function of *𝜖*. For comparison, also the matching based on the un-relocated recall fixations, for which fewer gaze density peaks are within 4^∘^ of the peak for encoding fixations. For small *𝜖*, peak position in more images are kept the same, which demonstrates the effectiveness of the relocation mapping

The number of leftover encoding fixation depend on the matching radius *𝜖*, We can form a measure of the reduction rate: the number of leftover encoding fixations divided by the original number of encoding fixations. The graph in Fig. 11a depicts the number of matched encoding fixations as a function of the matching radius. As expected, when using a small *𝜖*, the matching leads to better preservation of encoding fixations and fewer leftover fixations.

If we would not relocate recall fixations, fewer encoding fixations would be matched. For *𝜖* = 1^∘^ nearly 20*%* of encoding fixations were matched to relocated recall fixations, whereas only 5*%* were matched to original, unrelocated recall fixations. Note that this effect was quite similar across different images and observers. For values larger than *𝜖* = 10^∘^, we effectively matched all encoding fixations.

We also analysed whether the matching procedure shifts the matched gaze density map away from the encoding map, as opposed to just uniformly reducing the number of fixations without shifting the resulting distribution (which would be a flaw of the algorithm). For this we identified in each image the region with the most fixations during encoding, based on the smoothed spatial histogram. We did the same for the matched encoding fixations, which are fewer, and considered that the region had shifted if the difference in spatial location exceeds 4^∘^. Figure 11b shows the resulting difference in matched encoding fixations as a function of the matching radius *𝜖*. As expected, for large matching radii, the distribution of encoding fixations that could be matched against relocated recall fixations have a distribution very similar to the distribution of encoding fixations that are matched against the original un-relocated recall fixations. For smaller radii, there was a shift in the gaze density distribution in about 30*%* of the images when matching was made against relocated recall fixations. Matching against the original recall fixations led to distribution shifts in 40*%* of the images.

**Fig. 12:**
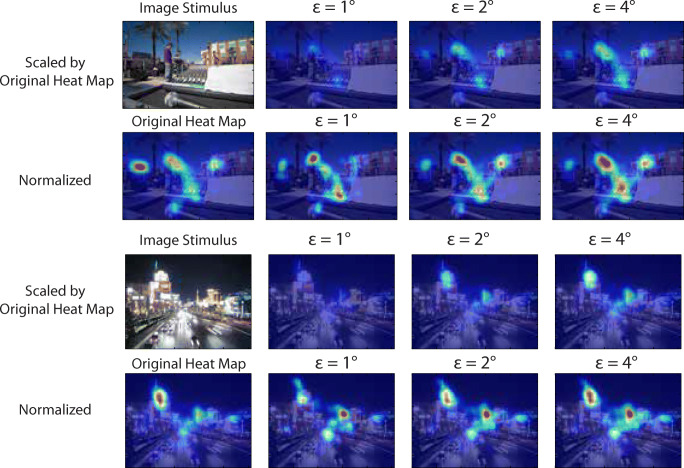
Matched encoding maps using different thresholds. We show examples of *𝜖* = 1^∘^,2^∘^,4^∘^. Original heat map shows the result based on all fixations during encoding of the image. The colour coding of the scaled maps is based on the highest value appearing in all heat maps, which is naturally the one generated without matching. They are used to show the resulting maps under the same scale. The normalized map is colour coded in its own range

Figure 12 illustrates the shifting effect from varying the threshold on the resulting gaze density maps for two of the images, for the values *𝜖* = 1^∘^,2^∘^,4^∘^. We can observe that some regions that attracted plenty of encoding fixations appear to have been less fixated during recall, for example the sign to the left in the top image. Other regions were more fixated during recall, such as the objects in the foreground in the top image or the tower to the right of the road in the bottom image. These examples show that the algorithm can indeed move the distribution when one part of the image is less present in recall fixations, while at the same time positioning recall fixations in what appears to be the right places.

We have shown that the exact parameter setting is not crucial for the result, as the method is reasonably stable across parameters. However, as is often the case for higher-order eye-movement methods, it can be advisable to run our matching algorithm with a range of parameters and make sure that the effect remains the same across parameter settings.

## Validation of the elastic consensus matching algorithm

Our choice of validation method was to record clicking data from participants who were asked to judge which is the most important region in each image. If our matching algorithm does a good job, we expect the resulting matched encoding fixations (based on relocated recall fixations) to fall on positions in the scene that were judged important in the clicking map.

### Do mapped recall fixations coincide with subjectively important scene elements?

**Fig. 13:**
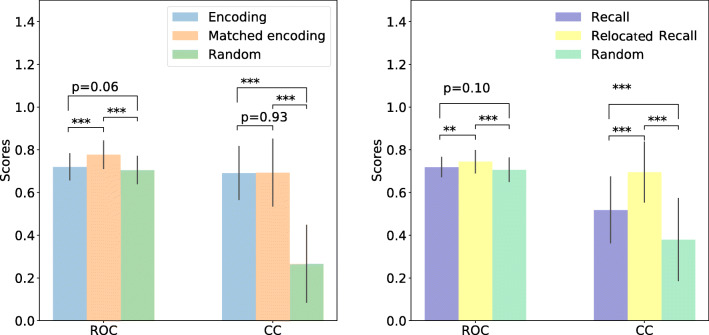
Averaged ROC and CC similarity scores. All comparisons are made against clicking maps. On the left, the blue bars compare gaze density maps during encoding (i.e. the encoding maps) to clicking (i.e. the clicking maps), while the orange bars compare the matched encoding fixations (i.e. the matched encoding maps) to clicking, and finally, the green bars compare the matched encoding maps based on random recall sequences to clicking. On the right, the blue bars instead compare gaze density maps during recall (i.e. the recall maps) to clicking, while orange bars compare the relocated and mapped recall results (i.e. the relocated recall maps) to clicking, and the green bars show the relocated recall maps when random encoding sequences are used for matching. Student t-test was used with sample size *n* = 100. ∗∗ is *p* < 0.005 and ∗∗∗ is *p* < 0.0005. Together the results indicate the proposed relocation algorithm is meaningful as the resulting gaze density maps have a higher correlation to clicking (importance), depicted by the orange and yellow bars

We first compared the similarity between clicking maps (such as Fig. 6) and encoding maps (i.e. gaze density maps from encoding fixations) versus matched encoding maps (i.e. gaze density maps from matched encoding fixations). Area under the ROC curve (Judd et al., 2012) and CC measures (Bylinskii et al., 2019; Holmqvist & Andersson, 2017) were used. Area under the ROC curve is a location-based metric which computes the trade-off between true and false positives under various thresholds. CC measures the linear correlation between two maps. We refer our readers to (Bylinskii et al., 2019) for computational details and a broader discussion on properties of several commonly-used metrics in the literature. The averaged similarity scores are plotted on the left in Fig. 13 where encoding maps (blue bars) and the the matched encoding maps (orange bars) were compared to clicking maps. Additionally, we also compared the clicking data to gaze density maps when randomly paired recall sequences were used in mapping (green bars).

We found a significantly smaller similarity with clicking maps when random sequences were used compared to the matched encoding maps (*t*(198) = 8.75,*p* ≪ 0.01 using ROC and *t*(198) = 19.84,*p* < .01 using CC, two-tailed student t-test).

As shown on the right part in Fig. 13, we then conducted similar comparisons between clicking maps and the recall maps (i.e. gaze density maps from original un-relocated recall fixations) (purple bars) versus the relocated recall maps (i.e. gaze density maps from relocated recall fixations) (yellow bars). Light green bars on the right show the similarities between clicking maps and the gaze density maps of the relocated recall fixations when randomly selected encoding sequences were used for relocation mapping. We observed significant decreases in matching from randomly paired sequences comparing to matching between the corresponding pairs (*t*(198) = 4.69,*p* ≪ 0.01 using ROC and *t*(198) = 13.02,*p* ≪ 0.01 using CC, two-tailed student t-test).

The larger similarity between clicking and recall fixations that have been relocated (yellow bars on the right) as well as the matched encoding fixations (orange bars on the left) suggests that the proposed matching algorithm produces a meaningful outcome: The areas clicked as important are largely equal to the matched encoding fixations produced by the algorithm, which is a correct behaviour if recall fixations during looking-at-nothing primarily correspond to the same important image regions as clicking maps. Note that the differences in the formulations of the used metrics may also contribute to the observed results. The ROC metric is most sensitive to the similarity between the peaks of two maps while the CC score measures pixel-wise correlation between two maps.

## Examples of encoding maps vs matched encoding and clicking maps

In this section, we report examples from our data set to give a concrete impression of how the algorithms works in practice, and what questions it can be applied for. Please note that these examples are not the result of experimentally controlled studies but taken as illustrative examples from our data set.

### Example 1: Increased entropy but not central bias

**Fig. 14:**
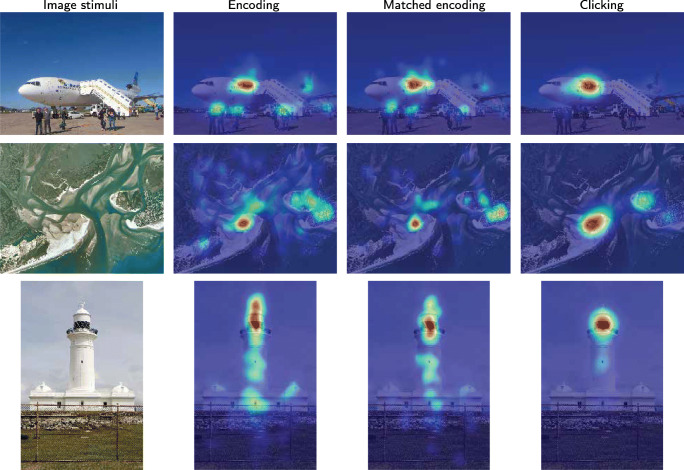
Examples of photographic images, gaze density maps for original encoding, for matched encoding based on relocated recall, and clicking data. Three examples are chosen such that the differences of the ROC scores are the largest, the median and the smallest

Figure 14 shows encoding maps vs matched encoding and clicking maps for three example photos. In all three cases, the peak - the most prioritized area - is the same. It can also be seen that peaks do not appear just in the center of the images, which would be the case if central bias Tatler (2007) would determine the choice of location in the matched encoding maps.

However, it can be clearly seen that during encoding, there is more gaze outside the top peak, than during matched encoding (based on relocated recall fixations). In the clicking map, the activity outside the peak has fallen almost to zero. Entropy increases, but does not give rise to central bias. It is the most interesting part of the photo that is ever more in focus in the matched encoding maps and clicking maps.

### Example 2: Low-level features

**Fig. 15:**
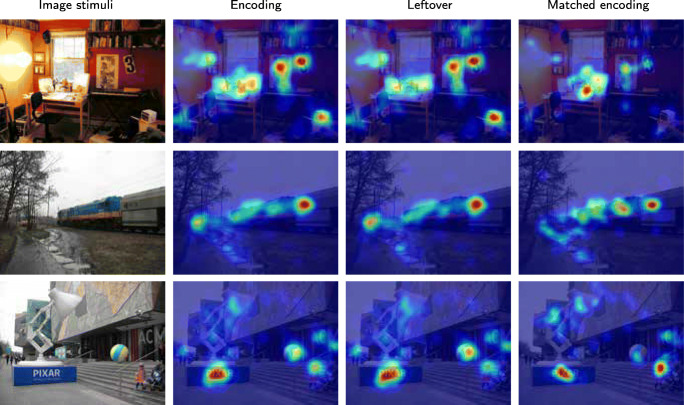
Examples of how low level features are not prioritized in recall. The second column shows the encoding map of the image. Leftover map shows the *map of non-prioritised objects* (very loosely “map of forgotten things”), while matched encoding fixations are used to generate the *matched encoding map* (very loosely “map of remembered things”). Each map is normalized in its own range. Fixations triggered by low-level features are largely absent in the *matched encoding* maps

Low-level features have been shown to contribute to saccade target selection (Badcock et al., 1996; Krieger et al., 2000; Kienzle et al., 2009). Consequently, low-level features contribute significantly to fixation-based attentional models (Itti and Koch, 2000, e.g). Figure 15 shows three examples of un-prioritized / forgotten scene elements. A white box in the lower right corner over a black background in the first image draws a lot of encoding fixations, possibly due to its high contrast. This box is less dominant in the resulting heat maps as shown in the last columns. The wall sign with the number 3 has a similar fate. In the second example, the red car behind the trees is large gone in the matched encoding fixations, in favour of fixations on the train. In the third example, the colourful ball is much less fixated during recall, but the little girl became relatively more fixated.

**Fig. 16:**
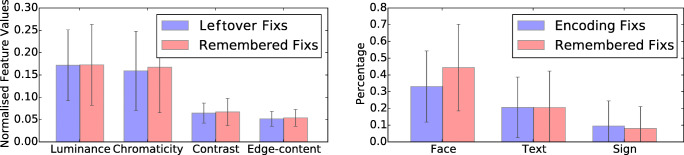
Left: The comparison of low-level feature values at fixated positions. Compared low-level features are luminance, chromaticity, contrast and edge-content. No difference between leftover fixations and matched encoding fixations (i.e. the remembered fixations) can be observed. Right: The percentage of fixations on faces, texts and signs in the encoding phase, and the corresponding number of matched encoding fixations (i.e. the remembered fixations) on the same objects. For faces, the p-value is 0.09

To quantify this effect in our non-experimental stimuli, we therefore calculated the feature values for luminance, chromaticity, contrast and edge-content following Tatler et al., (2005). As shown on the left in Fig. 16, no differences in low-level feature values could be found between positions of the leftover fixations during encoding and the matched (the remembered) fixations. The equal distribution of low-level features in leftover and matched encoding fixations may suggest that the prioritization in recall from episodic memory is not influenced by low-level features.

### Example 3: Faces, texts and signs

**Fig. 17:**
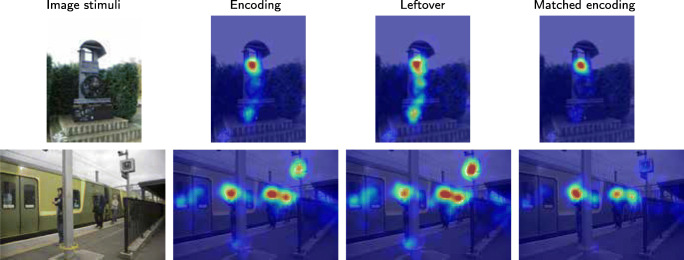
Text in the first example is entirely un-fixated during recall as shown in the last column, as well as the sign in the second example, where the people are fixated during recall, at the cost of other objects in the photos. Illegible or irrelevant text (upper row) as well as signs without particular relevance for the scene (lower row) are almost entirely un-fixated during recall

Many high-level (or semantic) features have been known to attract fixations during image inspection, most prominently faces but also signs and other artifacts as well as out-of-context objects. We have examples of high-level objects in our stimuli in the form of text, signs, and people. Figure 17 shows two examples. However, the variability in these uncontrolled photos made the comparison non-significant (Fig. 16 right).

To further examine the role of high-level features, we excluded the half of the participants whose recall fixations were less correlated to their encoding fixations, and then repeated the analysis for the remaining participants - with highly correlated fixations. The results showed a significant effect of not prioritizing text (*t*(54) = 2.83, *p* ≪ 0.05, two-tailed student t-test), indicating that text elements may not have a high recall priority once their meaning is decoded.

Additionally, we considered all features in each category regardless of their size. Size matters for attention, and may affect the prioritisation. In order to investigate the effect of size, we used the many differently sized faces in our diverse stimulus. Faces in photos are known to be fixation magnets, and hence also definite candidates for well-remembered objects in future controlled studies.

### Example 4: Big enough people are prioritised in recall and matched encoding fixations

**Fig. 18:**
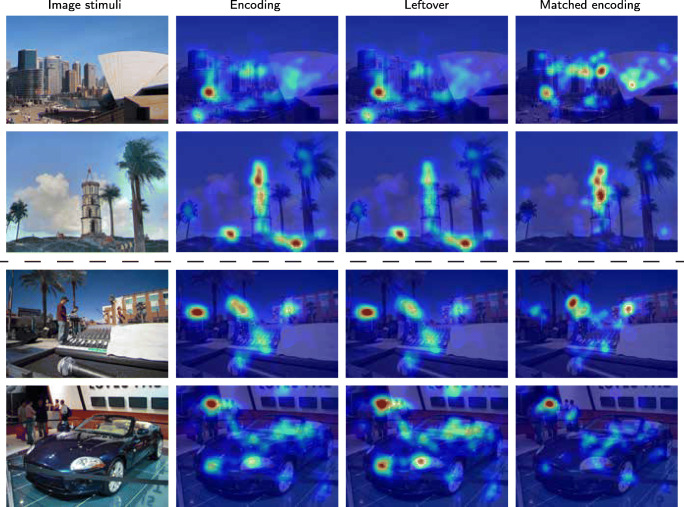
People in the scene are fixated during encoding but their reappearance during the recall fixation depends on their size in the photo. In the upper rows, the small people are not prioritized in recall, while in the lower rows they are big enough to be remembered. The meaning of the colour coded images is as before

**Fig. 19:**
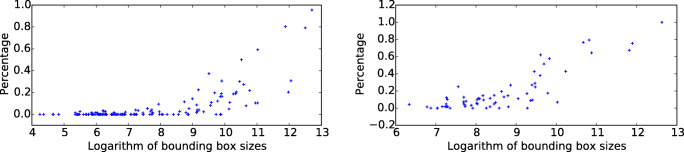
Left: the probability of a person being fixated during recall as a function of the size of the person in the photo. Small-sized people are forgotten, but at a certain extent, people are remembered. Right: The probability of a face being fixated during recall as a function of the size of the person in the photo. Faces covering a small area are forgotten, while larger faces are commonly recalled

There was a effect of size of people and faces on recall fixations. If a person was big enough, he or she would be fixated during recall, as in Fig. 18, usually at the cost of other things in the image. However, if people or faces were small in the photo, like in the top two rows of Fig. 18, they are largely un-fixated during recall. Figure 19 summarizes this size effect for all people and faces in the photos. In order to be fixated during recall of a photo, your body should fill around 2^∘^× 2^∘^ or more of the photo.

**Fig. 20:**
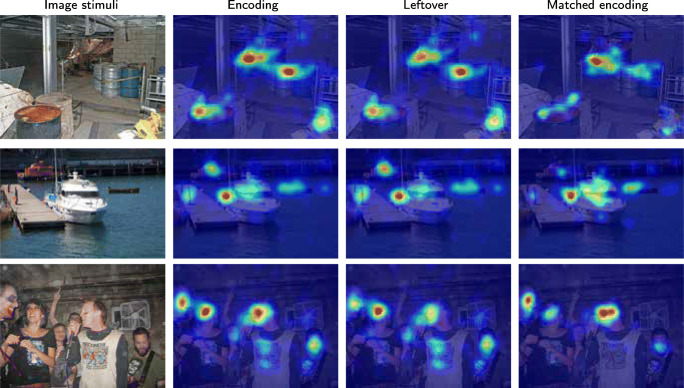
Effects on elements with similar meaning. The meaning of the colour coded images is as before. In the top row, similar distributions of encoding fixations among similar items are not preserved after matching against the recall fixations. The smaller boat in the middle row loses in relative priority during recall, while still being dominated by the larger boat. In the last row, the face that stands out among other faces increases in its dominance in the matched encoding map

Figure 20 exemplifies the fact that when several competing elements are side-by-side in the figure, the matched encoding often consists of a redistribution of gaze from some elements to others. The heatmaps in the matched encoding data should coincide with the elements prioritized during recall.

## Discussion

We have presented a novel algorithmic method for use with the looking-at-nothing (LAN) paradigm. Our elastic matching algorithm requires no specific parameter setting or fine-tuning, other than what we have presented in the description of the algorithm. We could show stable results across a wide range of parameters, which demonstrates the consistency of the matching algorithm in retrieving the underlying correspondences between fixations during encoding and subsequent recall. This algorithm is useful in variants of the LAN paradigm where the participants are silent during recall, i.e. when there is no participant speech to associate with recall fixations, and the only data from recall are fixations on an empty display. The matched encoding fixations can be expected to be close to the intended recall region of the photo, and can as such be taken to show what has been prioritized in episodic memory during LAN experiments.

When asked to click the most important position in the image, participants’ click positions consistently coincided with the peak of the gaze density map made from the matched encoding fixations based on relocated recall fixations. Indeed, our validation assumes that when participants choose to make silent recall fixations on an empty display, they prioritise to fixate positions where the important objects from the image would have been. We cannot know that this is what they actually do, i.e. that they have actually retrieved the important object from memory, but the consistent results we get nevertheless suggest that participants actually do prioritise these important image regions during recall.

The proposed method assumes that what is being prioritized in episodic memory has been fixated before, or in other words, that items not fixated during encoding cannot be recalled. This is contradicted by Underwood et al., (2003), who reported that of all the objects that were *not* fixated while participants watched video recordings of moving vehicles, nearly 20*%* were nevertheless recalled. When asked to describe pictures, participants often talk about objects that were never fixated before (Griffin and Spieler, 2006). This seems to be a natural limitation not only to the looking-at-nothing paradigm, but also of the method of eye-tracking in general. However, fixating in recall what was not fixated in encoding can be assumed to happen so rarely in the LAN paradigm that it should play no role for the results of the algorithm.

What exactly is encoded in visual episodic memory is of less importance for the algorithm to work, as long as this representation results in LAN eye movements suggestive of a mental image, as found by Johansson et al., (2006). Subjective importance, with whatever biases participants may have, will determine what is fixated, clicked, and encoded. If users of our algorithm want to control importance experimentally, it is up to those users of the algorithm to make decisions how to instruct participants on what is important, or to control stimulus images experimentally.

Central bias (Tatler, 2007) does not seem to explain our results. The entropy of fixation positions the MIT data set we used showed that the images generate a large variation in fixation position, and this variation can be clearly seen in the many examples of encoding, recall, clicking and matched encoding maps that we have presented.

In addition to utilising fixation position during recall, the duration of fixations could also be investigated. It is known that recall fixations are longer, but it is not known why. Possibly future experiments could attempt to tease out the role of fixation durations in recall by manipulating the objects to be recalled, for instance by how comment they are or how expected they are in the context, and investigate which manipulations lead to longer recall fixations. It is also unclear why the durations of recall fixations decrease over time, but it is possible to speculate that it might reflect a decrease in priority of the corresponding recall elements in episodic memory. The correlation between early recall fixations from the matching algorithm and clicking on the most important image region is 0.678, while correlation between late recall fixations based matching and clicking is 0.642 (t(198)= 1.75, p = 0.08, two-tailed student t-test), which may suggests that such a tendency could exist.

Our algorithm is not a scanpath comparison algorithm like MultiMatch, SubsMatch, Scanmatch etc (Dewhurst et al., 2012; Kübler et al., 2017; Anderson et al., 2015). Our algorithm entirely ignores the sequence order of fixations, as the general consensus is that sequence information from encoding is not repeated in recall. Furthermore, our algorithm is based on the assumptions that there are usually more fixations in encoding than in recall, and that the encoding fixations are the true and static positions to which we will move the erroneously located recall fixations. Our algorithm does not aim to establish a distance between the two sets of fixations, it just maps one set onto the other. That said, some scanpath comparison methods have components that just map one set of fixations onto another, and our method resembles those components, such as the mapping stage in MultiMatch

## Conclusions & Outlook

The looking-at-nothing paradigms offers many possibilities for research on encoding into and recall from visual episodic memory. When coupled with our matching function for encoding vs. recall fixations, it reveals the fixated content that is spontaneously prioritized in recall from episodic memory. Examples of such prioritization show interesting results that differ from the prioritization during image encoding. The proposed matching function can be used to study eye movements during mental imagery in general, and it also extends the possibility of using the looking-at-nothing paradigm in specific applications.

## Footnotes

1 http://cybertron.cg.tu-berlin.de/xiwang/mental_imagery/em.html

2 Source code can be found on http://cybertron.cg.tu-berlin.de/xiwang/mental_imagery/em.html
